# Comprehensive evaluation of the efficacy and safety of LPV/r drugs in the treatment of SARS and MERS to provide potential treatment options for COVID-19

**DOI:** 10.18632/aging.202860

**Published:** 2021-04-20

**Authors:** Liusheng Wu, Yuzhen Zheng, Jixian Liu, Ruixing Luo, Dingwang Wu, Pengcheng Xu, Da Wu, Xiaoqiang Li

**Affiliations:** 1 Peking University Shenzhen Hospital, Clinical College of Anhui Medical University, Shenzhen 518036, Guangdong, China; 2 Department of Thoracic Surgery, Peking University Shenzhen Hospital, Shenzhen 518036, Guangdong, China; 3 Anhui Medical University, Hefei 230032, Anhui, China; 4 Department of Thoracic Surgery, The Sixth Affiliated Hospital, Sun Yat-Sen University, Guangzhou 510655, Guangdong, China

**Keywords:** COVID-19, SARS, MERS, LPV/r, meta-analysis

## Abstract

Coronavirus disease 2019 (COVID-19) experienced an outbreak that expanded worldwide. Lopinavir/ritonavir (LPV/r), which is used effectively for severe acute respiratory syndrome (SARS) and Middle East respiratory syndrome (MERS) coronavirus infections, was applied for COVID-19 treatment given similarities in the molecular structures of these viruses. We performed a systematic review and meta-analysis to evaluate the efficacy and safety of lopinavir/ritonavir antiviral treatment in patients with SARS, MERS, and COVID-19. After registration with INPLASY, a search was conducted in PubMed, Embase, China National Knowledge Infrastructure (CNKI), Cochrane Library, WanFang Data, China Biomedical Literature Database (CBM) and other databases for all relevant literature on lopinavir/ritonavir treatment of SARS, MERS and COVID-19. The Cochrane Collaboration’s bias risk assessment tool and the Newcastle-Ottawa Scale (NOS) were used to evaluate the quality of the literature, and RevMan 5.3 software was used to evaluate the relevant outcome indicators of the efficacy and safety of lopinavir/ritonavir in the treatment of COVID-19. A total of 18 eligible studies (including randomized controlled studies, cohort studies, and case-control studies) were retrieved and included with a total of 2273 patients. The lopinavir/ritonavir group exhibited an increased nucleic acid conversion rate (P=0.004), higher virus clearance rate (P<0.0001), lower mortality rate (P=0.002), and reduced incidence of acute respiratory distress syndrome (ARDS) (P=0.02) compared with the control group. No significant benefit in the improvement rate of chest CT (P=0.08) or incidence of adverse events (P=0.45) was noted. The lopinavir/ritonavir group had a lower incidence of acute respiratory distress syndrome (P=0.02). According to the clinical prognostic results, the incidence of adverse events between the two groups was not statistically significant (P<0.0001). The efficacy of lopinavir/ritonavir in the treatment of patients with SARS, MERS and COVID-19 was significantly better than that of the control. Furthermore, the incidence of adverse events did not significantly increase. Lopinavir/ritonavir is effective in the treatment of COVID-19, and this combination should be further assessed in RCT studies. In addition, when we analyzed the differences in age and sex, we found that the differences were statistically significant in the safety and effectiveness of lopinavir/ritonavir in patients with COVID-19, and both of these factors played a significant role in the trial.

## INTRODUCTION

In December 2019, a new type of coronavirus named severe acute respiratory syndrome coronavirus 2 (SARS-CoV-2) caused an outbreak, and it quickly expanded to all over the world. SARS-CoV-2 is a single-stranded, positive-sense RNA virus that belongs to the Orthocoronavirus subfamily of the kingdom Coronaviridae of the order Nidovirales [1–3]. The Orthocoronavirus subfamily can be divided into four genera: α, β, γ and δ. SARS-CoV-2 belongs to the β genus [4]. This virus is a coronavirus that can infect humans and is similar to Middle East respiratory syndrome coronavirus (MERS-CoV) and severe acute respiratory syndrome coronavirus (SARS-CoV). The SARS-CoV-2 genome is similar to that of SARS-CoV and MERS-CoV and exhibits 70% and 40% sequence homology with these two viruses, respectively. Some researchers [5–7] used computer molecular docking methods to determine that SARS-CoV-2 and SARS-CoV share certain similarities in the molecular pathways of infected people. Therefore, a summary of previous anti-infective treatment research for SARS and MERS is conducive to identifying an effective treatment for COVID-19. However, the efficacy and safety of many antiviral drugs differ clinically, and COVID-19 treatment has introduced great difficulties.

The SARS outbreak occurred in 2002 and spread to Southeast Asia and the world. It was a global infectious disease epidemic [8] that was gradually eliminated by mid-2003. SARS-CoV has a similar molecular structure to that of SARS-CoV-2, which broke out and spread globally in the beginning of 2020 [9]. Therefore, previous SARS clinical treatment-related research [10] was summarized, analyzing a variety of effective treatment methods and evaluating the efficacy and safety of therapeutic drugs for guiding significance to improve the efficacy of SARS-CoV antivirals. Relevant studies [11–13] have performed clinical bioinformatics analyses on SARS-CoV transcriptome data, explored the mechanism of immune damage omics, collected SARS-CoV transcriptome data in the public gene expression database (Gene Expression Omnibus, GEO) and screened differential genes [14]. Collective analysis and protein interaction analysis were used to explore the mechanism of immune damage related to SARS-CoV infection and apply a precision treatment platform to predict potential therapeutic drugs. Among these drugs, the antiviral drug combination LPV/r achieved good efficacy in the clinical treatment of SARS-CoV, was predicted as a targeted therapeutic regimen and provides a reference for the clinical treatment of COVID-19.

MERS-CoV appeared in Saudi Arabia in 2012 [15]. The epidemic mainly occurred in Middle Eastern countries and South Korea and was occasionally noted in other countries and regions. During the past decade, domestic and foreign medical scientists have conducted a considerable amount of new drug development research for SARS and MERS. To accelerate the discovery of potential treatment options for COVID-19, researchers can learn from experiences with SARS-CoV and MERS-CoV drug development. Based on anti-SARS-CoV and MERS-CoV drug research, research and development costs can be reduced, and the development cycle can be shortened as much as possible. In addition, statistical analyses on related drug clinical trials of MERS antiviral therapy were conducted to evaluate the efficacy and safety of the drug and accumulate information for the treatment of current patients with COVID-19.

Lopinavir/ritonavir (LPV/r, Kaletra® or Aluvia®) is a protease inhibitor combination developed by AbbVie in the United States and was approved by the US Food and Drug Administration in 2000 to treat human immunodeficiency virus type 1 (HIV-1). Although the main indication of LPV/r is HIV-1 infection [16], clinical data [17] demonstrate that LPV/r can significantly reduce the mortality of patients infected with SARS-CoV, and this drug has cured patients infected with SARS-CoV and MERS-CoV in previous case reports. LPV/r is also the first anti-HIV-1 drug reported to be used in the clinical treatment of SARS-CoV-2 infection [17, 18].

However, the efficacy and safety of LPV/r in the treatment of patients with COVID-19 are very controversial. Cao et al. [19] reported that LPV/r has poor therapeutic efficacy in patients with severe COVID-19, and there are serious complications, such as ARDS. In addition, numerous clinical trials of LPV/r in the treatment of patients with early, mild COVID-19 have shown good therapeutic effects, and the results indicate that the incidence of adverse events was not statistically significant. In addition, there are significant differences in the efficacy and safety of LPV/r treatment in patients of different races and different ages and even in patients with different underlying diseases (including diabetes, HIV infection, and hepatitis B infection) [20, 21]. Therefore, to solve the abovementioned problems, a meta-analysis was conducted to evaluate the clinical research objectively and systematically on LPV/r in the treatment of SARS, MERS and COVID-19 and comprehensively evaluate the efficacy and safety of LPV/r. This study provides guidance on the antiviral treatment of COVID-19.

In clinical trials of many drugs, researchers consider many factors. The patient age range is often set to 18 to 65 years, which limits the inclusion of elderly people (>65 years old) and also leads to the loss of the opportunity to study the different effects based on aging populations in drug trials.

Mikhail V Blagosklonny claim that mortality increases exponentially with age, which is the strongest predictor of mortality in COVID-19 [22]. Mortality is higher in men compared with women because men age faster, and it is especially high in patients with age-related diseases. These diseases are manifestations of aging and a measure of biological age.

Camillo Sargiacomo [23] published a study on COVID-19 and chronological aging in the journal *Aging-US*, and he hypothesized that anti-aging drugs would be effective for the treatment or prevention of coronavirus infection. Therefore, he suggests that the fight against COVID-19 disease should involve testing the hypothesis that anti-aging drugs may have a prominent role in preventing the transmission of the virus.

## MATERIALS AND METHODS

### Literature search strategy

A request to register the present systematic review was submitted to INPLASY (INPLASY22080007) on July 20, 2020. The two researchers independently conducted systematic literature searches on PubMed, Cochrane Library, Embase and clinical trial registry platforms (http://clinicaltrials.gov/and http://www.chictr.org.cn/). Trials from the establishment of the database to July 2020 were included. The document language was limited to English and Chinese. We set the search keywords as follows: ("COVID-19" OR "SARS-CoV-2" OR "SARS-CoV" OR "MERS-CoV") AND ("lopinavir/ritonavir" OR "LPV/r"). Article types included randomized controlled trials (RCTs), cohort studies, and case-control studies. The references of the included studies and review articles were also reviewed to identify additional relevant studies.

### Inclusion criteria and exclusion criteria

① Participants: patients with positive nucleic acid test results for SARS, MERS or COVID-19; ② Intervention method: use of LPV/r for antiviral therapy; ③ Controlled study: use of other antiviral drugs or lack of use of LPV/r for antiviral therapy; ④ Research outcome: virus nucleic acid conversion rate, chest CT improvement rate, virus clearance rate, mortality rate, incidence of adverse events (AE), etc. ⑤ Research type: randomized controlled trials (RCTs), cohort studies, and case-control studies.

### Quality evaluation and data extraction

Two investigators independently evaluated the quality of the trials and extracted data. According to the standards in the Cochrane Handbook for Systematic Reviews of Interventions, the quality of the trial was evaluated based on the following aspects: selection bias, implementation bias, measurement bias, follow-up bias, reporting bias and other potential sources of bias. The extracted content was as follows: ① basic information of the article, such as author and publication year; ② research design: treatment path, treatment plan, number of patients in each group, administration method, dominant race, etc.; ③ observation results: viral nucleic acid conversion rate, chest data indicators, CT improvement rate, virus clearance rate and mortality rate and 95% CI, OR, RR and AE incidence. The two researchers discussed and resolved their differences. If they could not reach an agreement, the third researcher decided.

### Data extraction

Stata 12.0 (Stata Corp, College Station, TX, USA) and RevMan 5.3 (The Nordic Cochrane Centre, Copenhagen, Denmark) were used to calculate the relative risk (RR) and 95% CI of ORs and AEs. Egger’s linear regression analyses and funnel plots were used to assess publication bias, and a Q test was used for heterogeneity analyses. The I^2^ statistic was used to assess the level of heterogeneity among studies. When I^2^ was > 50%, the heterogeneity was considered significant, and a random effects model was used for analysis. Otherwise, a fixed effects model was used. Subgroup analysis was used to investigate possible sources of heterogeneity: dominant race, administration method and treatment route. If heterogeneity could not be eliminated by the subgroup analysis, a sensitivity analysis was performed to further determine the source of heterogeneity. All P-values were two-sided tests. P<0.05 was considered statistically significant.

### Ethics approval

Ethics approval was not required for this systematic review.

## RESULTS

### Literature retrieval results and basic characteristics of the included studies

**Figure 1:**
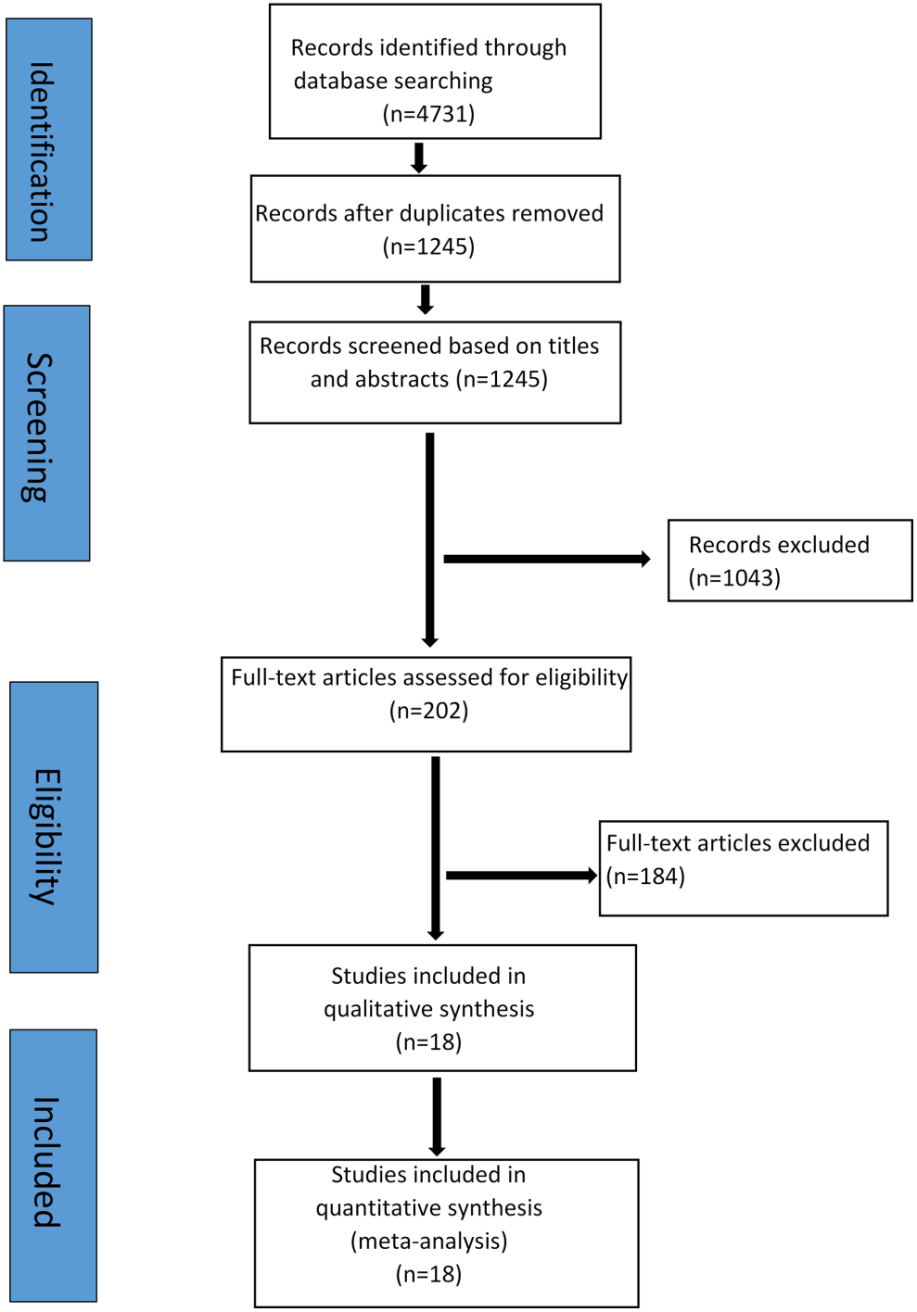
Flow chart of the literature search and study selection procedure.

The preliminary retrieval identified 4731 articles and excluded 3486 articles, including duplicates, irrelevant studies and articles with insufficient data. Among the remaining 1245 articles, 1043 articles, including case reports, basic research, and animal experiments, were excluded. Of the remaining 202 articles, 184 articles were eliminated by the Jadad score and NOS score for literature quality. Finally, 18 articles [19, 21, 22, 24–38] including 2273 patients were successfully included in the study (Supplementary Table 1). The literature screening process and results are shown in Figure 1, and the basic characteristics of the studies are shown in Supplementary Table 1.

Our study collected ongoing studies on LPV/r drug clinical trials (Supplementary Table 2). A total of 15 clinical trial studies on LPV/r for the treatment of COVID-19 included 6 single-drug regimens and 9 combination regimens. We found that an increasing number of trials have explored and evaluated LPV/r in combination with other drugs to treat COVID-19, providing increasingly effective options for patients with COVID-19.

### Risk of bias and quality of evidence assessment

**Figure 2:**
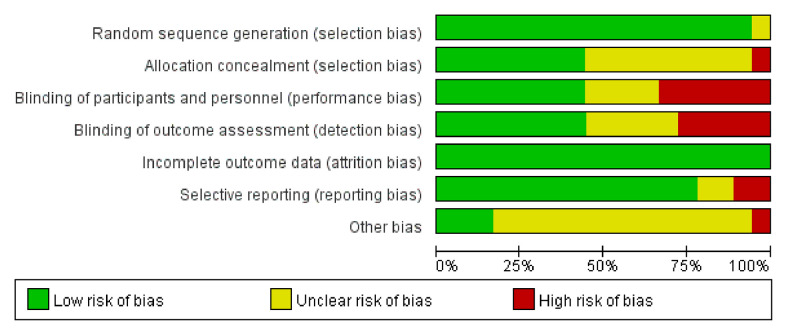
Risk of bias graph.

**Figure 3:**
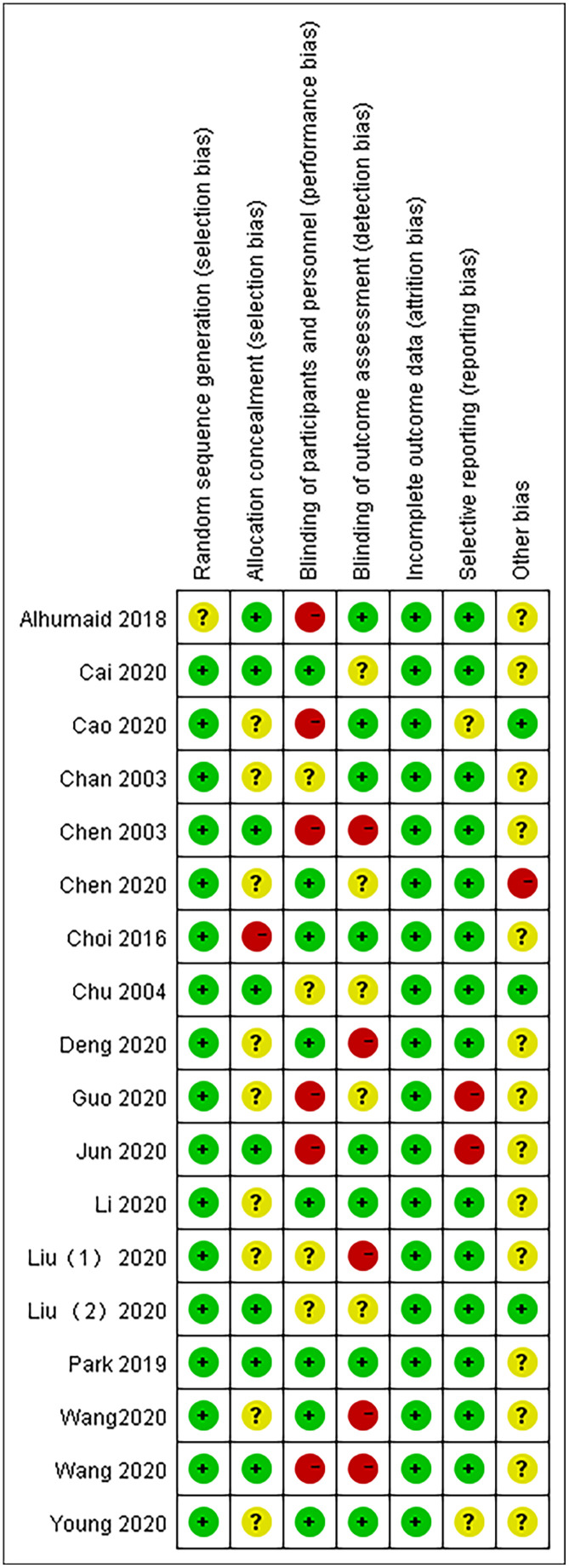
Risk of bias summary.

According to the Cochrane Systematic Review Manual (version 5.1.0), two investigators independently assessed the risk of bias in the included studies. The overall methodological quality of the 18 articles was good and fair. The GRADE working group evaluated the quality of evidence for each result. Mortality and adverse events (AEs) had a high level of evidence, the nucleic acid conversion rate had an intermediate level of evidence, and the virus clearance rate and chest CT improvement rate had a low level of evidence (Figures 2, 3).

Our study evaluated the quality assessment of 18 articles. We found performance bias of blinding of participants and personnel and detection bias of blinding of outcome assessment. Figures 2, 3 present the quality assessment of the literature in our studies. The green label indicates a low risk of bias, the yellow label indicates an unclear risk of bias, and the red label indicates a high risk of bias.

Based on the risk of bias graph, we observed a low risk of selection bias and attrition bias but still a high risk of performance bias in 18 studies.

From the risk of bias summary, we observe both the overall distribution of bias risk and the degree of bias risk in each research study. There may be concentrated in the overall risk bias, but it is a very small proportion in each individual study.

### Evaluation of the effectiveness of LPV/r in the treatment of SARS, MERS and COVID-19

### Nucleic acid conversion

**Figure 4:**
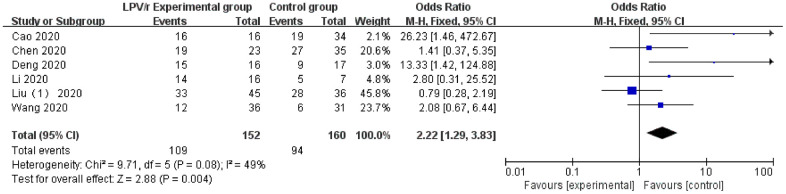
Forest plot of the meta-analysis of nucleic acid conversion.

A total of 6 studies [19, 29, 30, 34, 35] reported data on the nucleic acid conversion rate with LPV/r clinical treatment (the 6 trials were all COVID-19 studies). Egger’s test (P = 0.15) and the funnel plot did not demonstrate significant publication bias. The heterogeneity test revealed that there was no significant heterogeneity among the trials (I^2^ <50%), and the fixed effects model was used for statistical analysis. The results showed that the LPV/r experimental group [71.7% (109/152)] was significantly better than the control group without LPV/r or other antiviral drugs [58.8% (94/160)] in the treatment of COVID-19 [OR=2.22, 95% CI: 0.67~6.44, P=0.004] (Figure 4).

### Mortality rate

**Figure 5:**
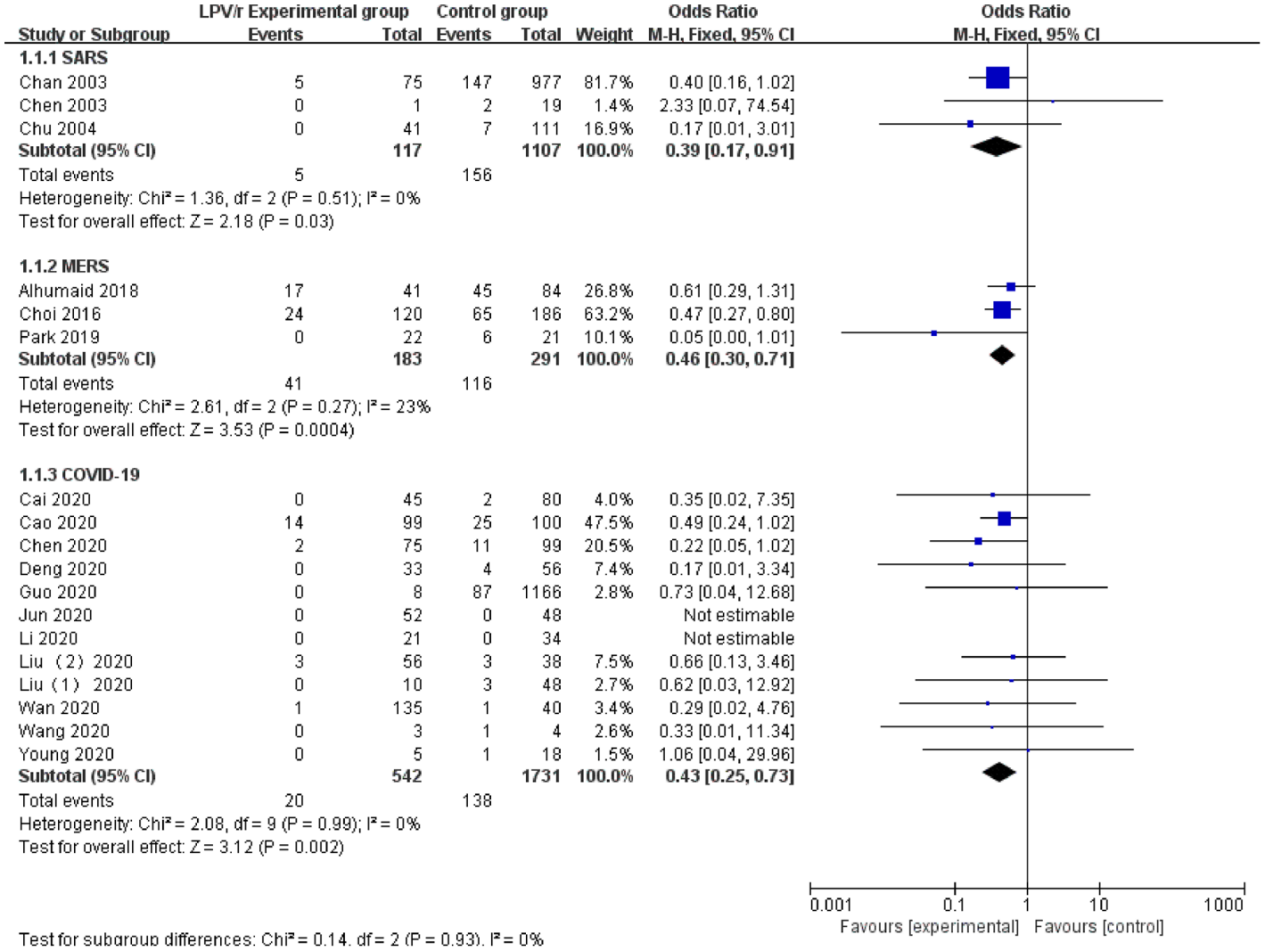
Forest plot of the meta-analysis of the mortality rate.

The 18 included articles had mortality data for LPV/r treatment (SARS: 3 articles, MERS: 3 articles, and COVID-19: 12 articles). Egger’s test (P = 0.084) and the funnel plot did not demonstrate publication bias. The heterogeneity test revealed no significant heterogeneity among the trials (I^2^=0%), and the fixed effects model was used for synthetic analysis. The results showed that the LPV/r experimental group [3.69% (20/542)] had significantly lower mortality in patients with SARS, MERS or COVID-19 compared with the control group [7.97% (138/1731)], and the clinical treatment effect was significantly better [OR=0.43, 95% CI: 0.25~0.73, P=0.002] (Figure 5).

### Virus clearance rate

**Figure 6:**
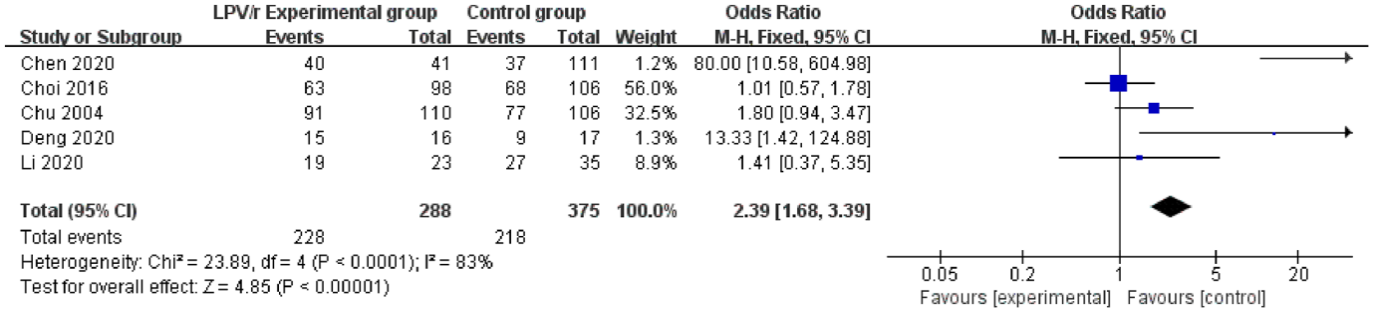
Forest plot of the meta-analysis of the virus clearance rate.

Five studies [22, 25, 29, 32, 36] reported data on virus clearance rates (SARS: 1 article, MERS: 1 article, and COVID-19: 3 articles). Egger’s test and the funnel plot did not demonstrate publication bias. The heterogeneity test revealed significant heterogeneity among the trials (I^2^=83%). To explore the source of heterogeneity, race, age and severity of the disease, which may have been related to the heterogeneity, were included in the study. A fixed effects model was used for synthetic analysis. The results showed that the LPV/r experimental group [79.17% (228/288)] had significant virus clearance in the antiviral clinical treatment of SARS, MERS and COVID-19 compared to the control group [58.13% (218/375)] [OR=2.39, 95% CI: 1.68~3.39, P<0.00001] (Figure 6).

### Chest CT improvement rate

**Figure 7:**
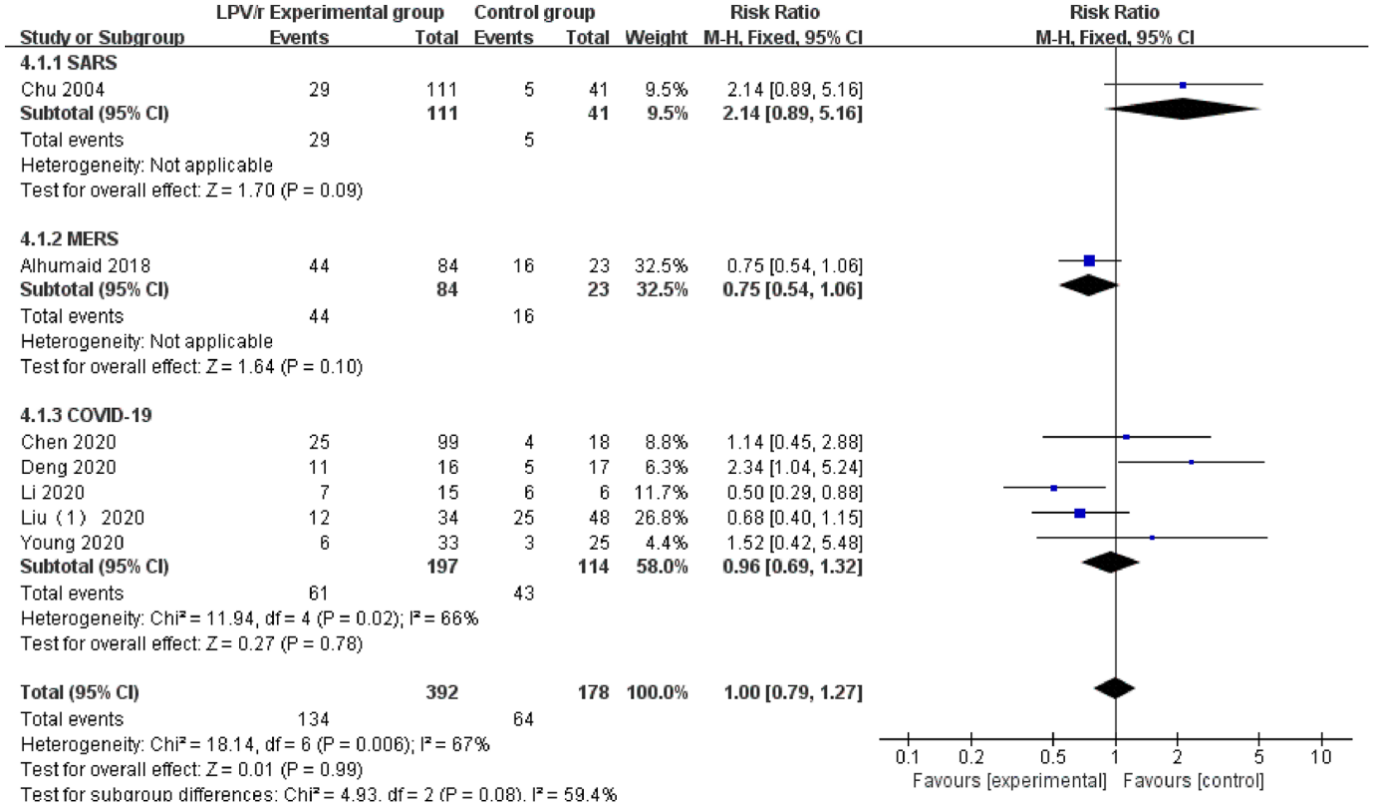
Forest plot of the meta-analysis of the chest CT improvement rate.

A total of 7 studies [22, 26, 28, 29, 32, 33, 36] reported relevant data on the chest CT improvement rate with LPV/r treatment (SARS: 1 article, MERS: 1 article, and COVID-19: 5 articles). Egger’s test (P = 0.084) and the funnel plot did not demonstrate publication bias. The heterogeneity test revealed significant heterogeneity (I^2^=67%) among the 7 trials. A fixed pattern was used to analyze the heterogeneity, and the source of the heterogeneity was traced. The source may differ from the underlying diseases (diabetes, HIV infection, hepatitis B infection, etc.). The results showed no statistically significant differences between the LPV/r experimental group and the control group [RR=1.00, 95% CI: 0.96~1.32, P=0.08] (Figure 7).

### Safety assessment of LPV/r treatment for SARS, MERS and COVID-19

### ARDS

A total of 4 studies [22, 25, 26, 29] reported data on ARDS for LPV/r treatment (SARS: 1 article, MERS: 2 articles, COVID-19: 1 article). The heterogeneity test demonstrated that there was no significant heterogeneity among the trials (I^2^=0%), and a fixed effects model was used for analysis. The results showed that the LPV/r experimental group [18.87% (47/249)] effectively reduced the incidence of ARDS compared with the control group [40% (34/85)]. The safety assessment was high, and the difference was statistically significant [OR=0.46, 95% CI: 0.25~0.87, P=0.02].

### Adverse events (AEs)

A total of 6 studies [30, 31, 33–35, 38] reported adverse events (AEs) during LPV/r treatment (COVID-19: 6 articles). Egger’s test and the funnel plot did not demonstrate publication bias. The heterogeneity test revealed no significant heterogeneity among the trials (I2=0%), and a fixed effects model was used for analysis. The results showed that the difference between the LPV/r experimental group [26.81% (85/317)] and the control group [27.48% (97/353)] was not statistically significant [OR=0.87, 95% CI: 0.60~1.25, P=0.45].

### Intubation and mechanical ventilation

A total of 3 studies [24, 25, 27] reported data on intubation and mechanical ventilation in LPV/r therapy (SARS: 1 article and MERS: 2 articles). The heterogeneity test demonstrated no significant heterogeneity among the trials (I^2^=0%), and a fixed effects model was used for analysis. The results showed that the LPV/r experimental group [29.53% (44/149)] had a higher intubation rate and proportion of mechanical ventilation compared with the control group [4.27% (29/679)], but the difference was not statistically significant [OR=0.60, 95% CI: 0.27~1.34, P=0.22].

### Leukopenia

A total of 5 studies [25, 26, 29, 32, 38] reported data on leukopenia in LPV/r antiviral therapy (MERS: 2 articles and COVID-19: 3 articles). The heterogeneity test demonstrated significant heterogeneity among the trials (I^2^=67%), and a fixed effects model was used for analysis. The results showed no statistically significant differences between the two groups [OR=0.83, 95% CI: 0.53~1.29, P=0.40].

### Anemia

A total of 4 studies [28, 34, 35, 37] reported data on the occurrence of anemia with LPV/r antiviral therapy (COVID-19: 4 articles). The heterogeneity test demonstrated significant heterogeneity among the trials (I^2^=85%), and a fixed effects model was used for analysis. The results showed that the incidence of anemia in the LPV/r experimental group [39% (126/323)] was significantly greater than that in the control group [15% (45/300)], suggesting that there are complications of anemia when using LPV/r for antiviral therapy. The difference between the two groups was statistically significant [OR=3.45, 95% CI: 2.30~5.17, P<0.001].

### Thrombocytopenia

**Figure 8:**
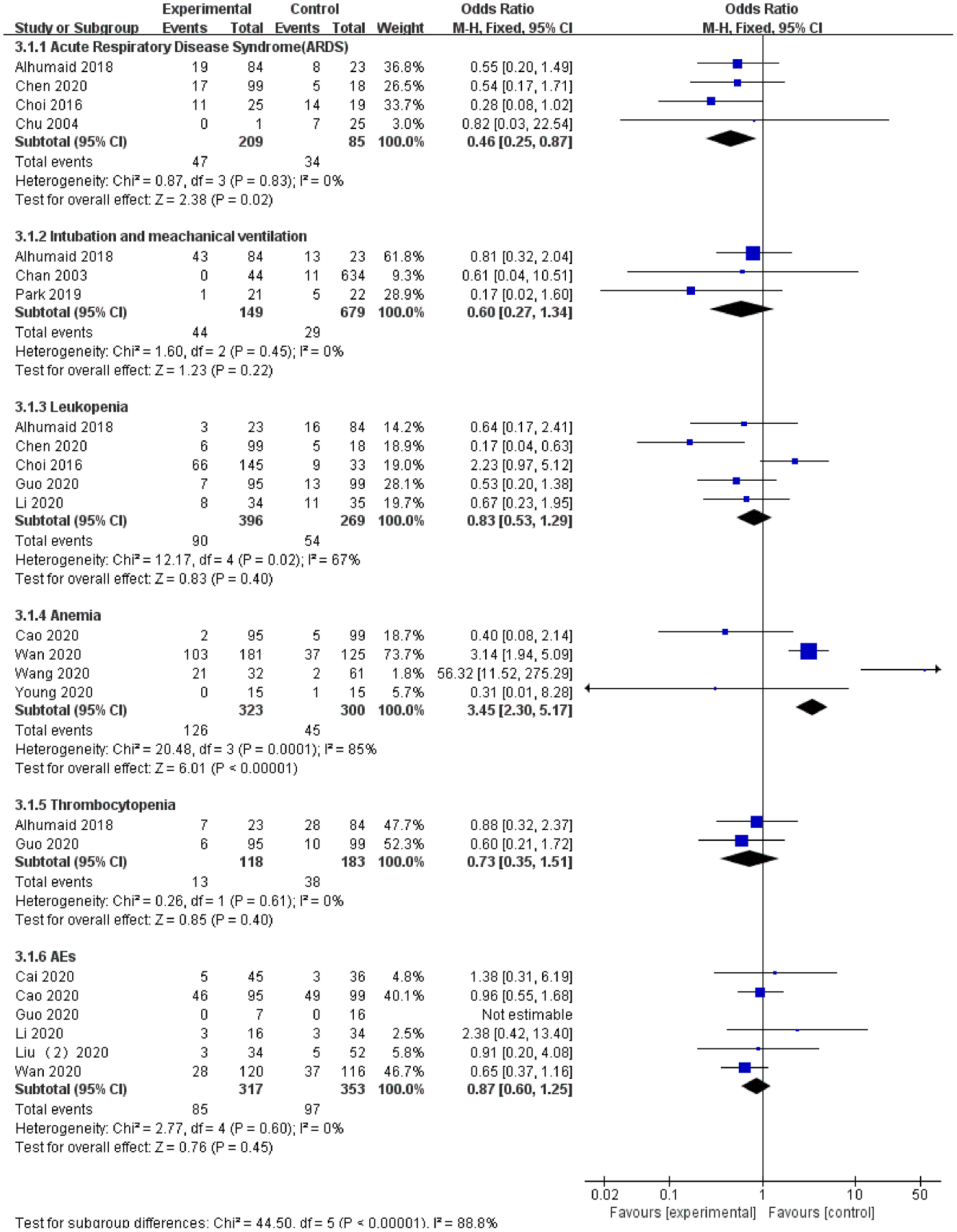
Forest plot of the meta-analysis of LPV/r drug safety assessment.

A total of 2 studies [26, 38] reported the occurrence of thrombocytopenia with LPV/r antiviral therapy (MERS: 1 article and COVID-19: 1 article). Egger’s test and the funnel plot did not demonstrate publication bias. The heterogeneity test revealed no significant heterogeneity between the trials (I^2^=0%), and a fixed effects model was used for analysis. The results showed that the difference between the two groups was not statistically significant [OR=0.73, 95% CI: 0.35~1.51, P=0.40] (Figure 8).

### Age-based differences in LPV/r therapy in patients with COVID-19

We collected and organized the clinical data of COVID-19 patients treated with LPV/r as much as possible in the 18 RCTs of our study and separately analyzed the available age data of patients. There were two groups: the elderly patient group (age> 65 years) and the nonelderly patient group (age <65 years). In addition, we also added the influencing factors of cardiopulmonary disease to analyze the role and effect of aging in the LPV/r treatment of COVID-19, and the data indicated that aging plays a significant role in LPV/r treatment of COVID-19.

In terms of efficacy, in the application of LPV/R against COVID-19, the nonelderly patients group exhibited more advantages than the elderly patients group in terms of efficacy: nucleic acid conversion (OR=0.20, 95% CI:0.13~0.32, P<0.00001), mortality rate (OR=5.57, 95% CI:1.63~19.08, P=0.006), and virus clearance rate (OR=0.37, 95% CI:0.18~0.76, P=0.007). However, in terms of the chest CT improvement rate (OR=1.19, 95% CI: 0.59~2.41, P=0.63), no significant difference was noted between the two groups (P>0.05).

In terms of safety, in the nonelderly patient group and the elderly patient group, more distinctly different complications were noted after treatment with LPV/r, and the difference was statistically significant (P<0.05).

**Figure 9:**
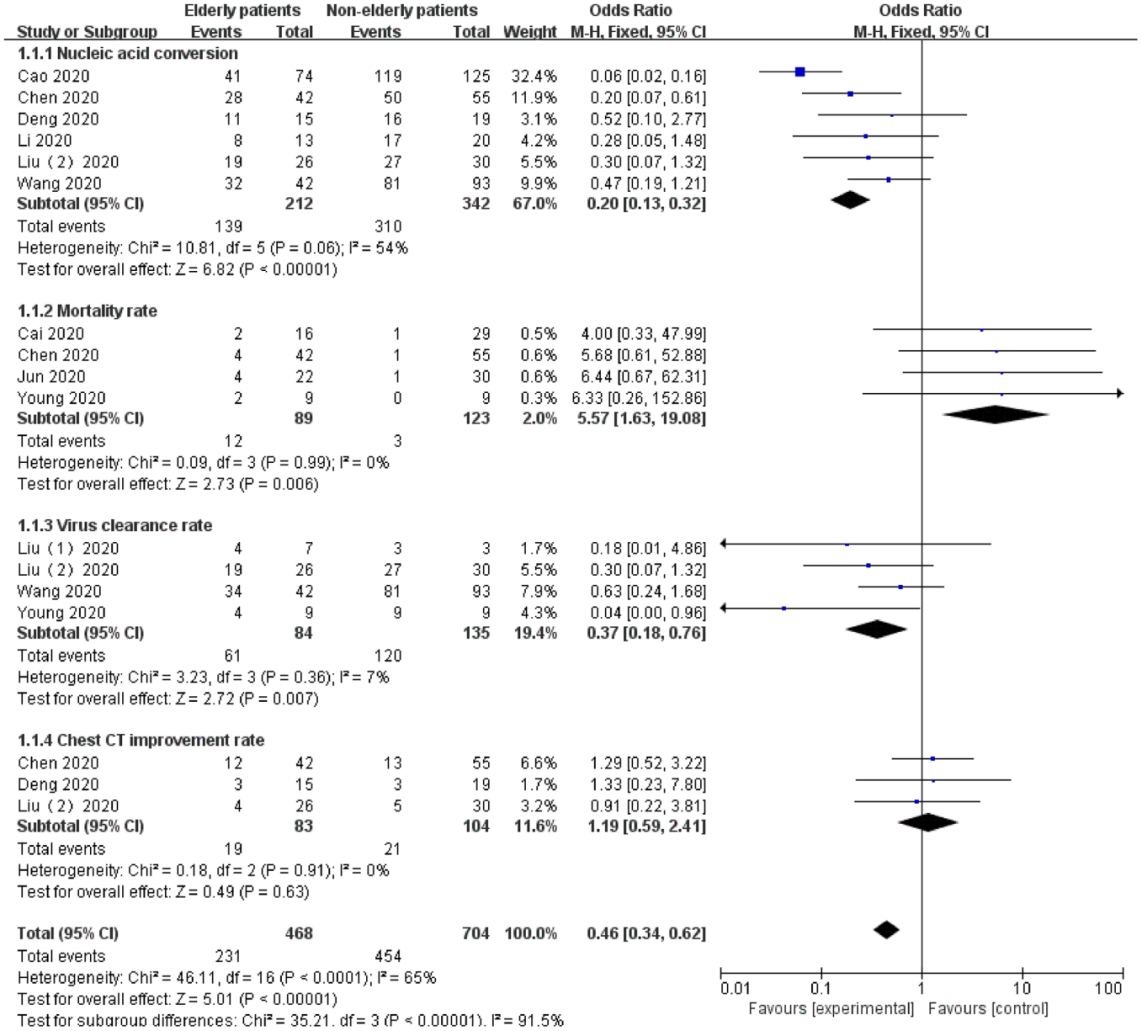
Forest plot of the meta-analysis of age-based differences in effectiveness in LPV/r therapy in patients with COVID-19.

The data showed that compared with the nonelderly patients group, the elderly patients group treated with LPV/r was more likely to cause ARDS (OR=4.84, 95% CI:1.52~15.41, P=0.008) and AEs (OR=2.69, 95% CI:1.15~6.29, P=0.02). However, there was no significant difference in intubation and mechanical ventilation (OR=1.74, 95% CI:0.83~3.64, P=0.14), leukopenia (OR=1.31, 95% CI:0.47~3.67, P=0.61), anemia (OR=1.75, 95% CI:0.43~7.07, P=0.43) and thrombocytopenia (OR=1.34, 95% CI:0.53~3.39, P=0.53) (Figure 9).

### Sex-based differences in LPV/r therapy of patients with COVID-19

Our study analyzed the data of sex-based differences in the research projects, and we considered that some clinical projects have the following problems: 1. The establishment of the control group was not unified; 2. Partial data loss was noted in the research; 3. Inconsistent evaluation criteria were employed for efficacy indicators.

We previously reviewed many studies. We found that in the treatment of some HIV patients with LPV/r, some scholars use immune cells (mainly CD-4+ T lymphocytes) as the efficacy indicator of LPV/r, but few studies have reported sex differences in LPV/r in antiviral therapy. However, we analyzed the gender data of patients included in the study group. There was a significant difference in LPV/r in the antiviral process of COVID-19 patients, and the difference was statistically significant (OR=1.58, 95% CI: 1.28~1.95, P<0.0001).

**Figure 10:**
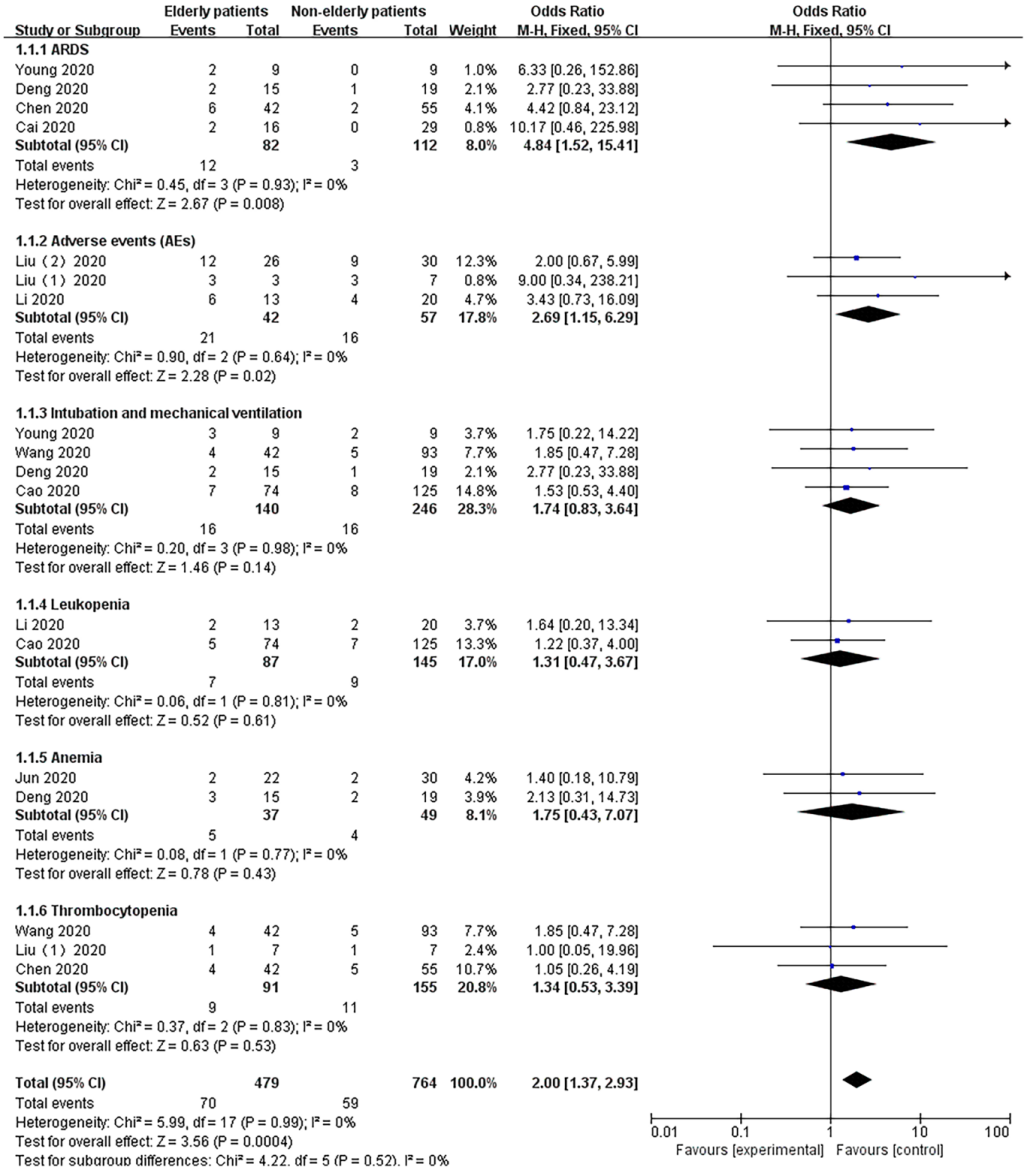
Forest plot of the meta-analysis of age-based differences in safety in LPV/r therapy in patients with COVID-19.

Some selection bias may also be noted. For example, in compliance with the randomized control principle, patients with COVID-19 were included in the study groups instead of following the strict selection criteria of a 1:1 male to female ratio, which may also have had an impact on the statistical results (Figure 10).

### Publication bias

The funnel plot of literature publication bias was symmetric, and all literature research experiments were controlled within the 95% confidence interval, indicating that there was no significant publication bias among the included studies.

**Figure 11:**
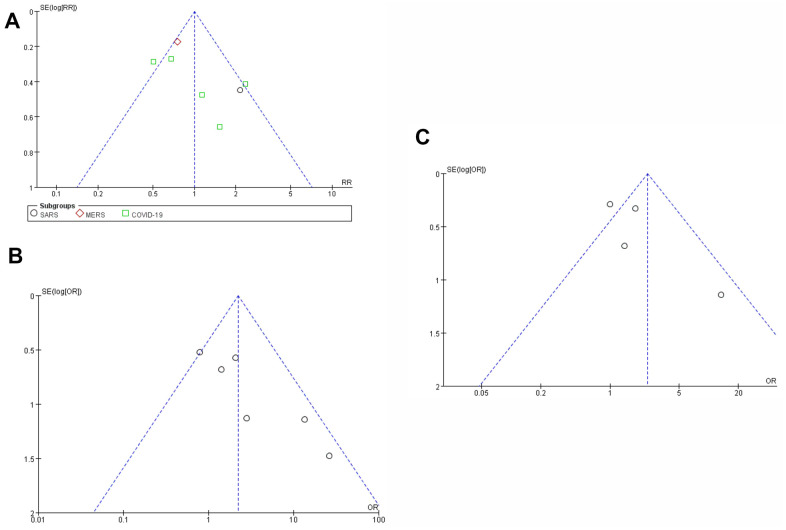
**Funnel plot of meta-analysis of publication bias.** (**A**) Funnel plot of publication bias for the efficacy and safety of SARS, MERS, COVID-19 and LPV/ r drugs. (**B**) publication bias test. (**C**) Trim and fill methods to test publication bias.

Taking the nucleic acid conversion rate of COVID-19 patients treated with LPV/R drug as an example, Begg’s test (P<0.001) and Egger’s test (P=0.989) indicated no obvious publication bias (Figure 11).

## DISCUSSION

LPV/r is widely used in the first-line treatment of respiratory virus infections and HIV infection. Some clinical trial research data show that LPV/r is effective against SARS-CoV, MERS-CoV and SARS-CoV-2. However, there are also some clinical trials that show that LPV/r has no effect on SARS, MERS and COVID-19. This study systematically integrated 18 relevant studies into a meta-analysis. The statistical results show that LPV/r exhibits an effect in patients with early mild SARS, MERS and COVID-19, and LPV/r did not increase the risk of adverse events. However, LPV/r had no effect in severe cases of SARS, MERS and COVID-19 and increased the risk of ARDS [19].

The active ingredients of LPV/r include LPV (200 mg) and low-dose RTV (50 mg) [7]. LPV/r was approved by the FDA in 2000 for the treatment of HIV-1 infection in adults and in 2008 for the treatment of HIV-1 infection in children over 2 years old. In China, the pharmacokinetics and clinical safety of LPV/r has been studied, and the drug combination was approved for the treatment of influenza and other respiratory virus infections [39–41]. The identification of an effective antiviral drug is a fundamental strategy in the treatment of coronavirus infections. Due to the urgent need for effective treatments of COVID-19, LPV/r has been increasingly repurposed for immediate use.

Although effective treatments for SARS-CoV-2 are currently lacking, treatment is based on the extremely high sequence homology between SARS-CoV-2 and SARS-CoV in the LPV/r target gene coding. This study analyzed whether LPV/r may represent an effective drug for the treatment of SARS-CoV-2. Clinical research data show that LPV/r is not effective in treating severely ill patients. This finding may be attributed to the fact that severely ill patients typically have abnormal liver function, and LPV can aggravate liver damage in the process of inhibiting liver metabolism. Another reason is that systemic hyperinflammation rather than viral pathogenicity dominates later stages of SARS-CoV-2. Therefore, other drugs are needed to treat critically ill patients.

We analyzed and evaluated the data of 18 clinical trials. These 18 studies reported mortality data for LPV/r treatment of SARS, MERS and COVID-19. According to the statistical analysis, the LPV/r experimental group exhibited a lower mortality rate than the control group. Five studies reported virus clearance. Seven studies reported the chest CT improvement rate. Six studies reported the viral nucleic acid conversion rate. The results of the statistical analysis showed a significant difference in the virus clearance and nucleic acid conversion rate between the two groups. However, we found no statistically significant differences in the chest CT improvement rate between the LPV/r experimental group and the control group. Few cases of SARS and MERS have been reported. Most cases in our study were COVID-19 cases. Chen et al. comprehensively studied LPV/r in COVID-19 cases and collected chest CT improvement data [29]. This study used a randomized controlled trial of the standard and LPV/r groups with 99 cases and reported 25 cases with lung CT improvement. Among the 18 cases in the control group that did not use LPV/r antiviral treatment, 4 cases demonstrated chest CT improvement. However, the difference was not statistically significant. Therefore, this study considered that LPV/r could not improve chest CT during antiviral treatment of SARS, MERS and COVID-19, which we hypothesize might be related to irreversible fibrosis of the lungs. Another consideration is that the evaluation of the efficacy of LPV/r cannot be reflected by the chest CT improvement rate.

We also assessed clinical prognosis outcome data for the LPV/r experimental group and the control group. Four studies reported cases of ARDS. Alhumaid et al. conducted a retrospective observational study of MERS with 19 cases of ARDS among 84 cases of MERS and 8 cases of ARDS among 23 patients in the control group [26]. Chen et al. conducted a randomized controlled study and reported 19 cases of ARDS in 99 cases of COVID-19 [29]. The difference was statistically significant. However, some studies included criteria for patients with mild disease, and the LPV/r drug curative effect was unclear in some patients with severe disease. Recently, Wang et al. (Beijing China-Japan Friendship Hospital) [19] published a clinical trial entitled "A trial of lopinavir–ritonavir in adults hospitalized with severe COVID-19" in the *NEJM*, which included an evaluation of the efficacy and safety of LPV/r. The results of this randomized controlled study suggest that LPV/r is ineffective in patients with severe COVID-19 and may even increase the risk of ARDS complications. This study provides valuable guidance for the first-line treatment of COVID-19. Hu et al conducted a study of the risk factors associated with clinical outcomes in 323 cases of COVID-19. Univariate analysis revealed that among patients receiving LPV/r (n=28), a higher proportion developed unfavorable outcomes, including death or disease progression (23.8% versus 5%, p<0.001), compared with those not receiving LPV/r (n=295). However, patients with critical disease at baseline were more likely to receive LPV/r than those with nonsevere and severe disease (p<0.001), indicating that the worse outcomes among those receiving LPV/r could be explained by bias [42].

A total of 6 studies reported adverse events, including nausea, vomiting, and fatigue; these symptoms can be relieved by later treatments and have minimal impact on the disease. Cai et al. conducted a randomized controlled trial with 95 patients in an LPV/r experimental group, and 46 cases of adverse events occurred [35]. In addition, 46 cases of adverse events occurred among the 99 cases in the control group. The statistical results showed no significant difference. Furthermore, both studies suggest that the use of LPV/r in patients with COVID-19 did not increase the risk of adverse events.

Although the WHO accepted the recommendation from the Solidarity Trial’s International Steering Committee to discontinue the trial’s LPV/r arms, significant findings in previous studies of lopinavir/ritonavir in the treatment of the virus should be analyzed. The recommendation is applicable to a population in hospitalized Solidarity Trial patients and is not applicable in other studies of LPV/r in nonhospitalized patients or as pre- or postexposure prophylaxis for COVID-19 (https://www.who.int/news-room/detail/04-07-2020-who-discontinues-hydroxychloroquine-and-lopinavir-ritonavir-treatment-arms-for-covid-19).

This paper extracted the latest LPV/r clinical experimental data for statistical analysis. This study not only evaluated the efficacy and safety of LPV/r treatment of COVID-19 but also retrospectively analyzed the previous SARS- and MERS-related LPV/r clinical trials for the treatment of three types of respiratory viruses and an in-depth study of the antiviral mechanism of LPV/r to provide guidance on the use of LPV/r in the treatment of the three viruses in the clinic and to evaluate the efficacy of LPV/r and emergency response to adverse events. Furthermore, the incidence of adverse events did not significantly increase. This study analyzed the similarities and differences between LPV/r treatments for three respiratory viruses, which has guiding significance for the clinical treatment and control of emergency programs for similar respiratory virus infections in the future.

To our knowledge, this study is the first systematic review and meta-analysis to comprehensively summarize all available evidence to evaluate the efficacy and safety of LPV/r in the treatment of SARS, MERS and COVID-19. The results of this study show that LPV/r may be safely used as a first-line antiviral treatment for COVID-19 in the future. It is of utmost importance to conduct large-scale and high-quality RCTs with high-quality research designs and more studies on the antiviral mechanism of LPV/r to develop better therapeutic regimens.

Relevant studies have reported that LPV/r demonstrates good clinical efficacy in SARS, MERS and COVID-19 and does not increase the risk of adverse events with similar antiviral drugs. Therefore, it is worthwhile to promote the use of LPV/r.

Our results may allow clinicians to comprehensively understand the properties of each anti-coronavirus agent in terms of efficacy and safety outcomes and thus may constitute a basis for drug treatment for COVID-19. It is important to conduct large-scale clinical trials to objectively assess the efficacy of antiviral treatment on the mortality and virological and clinical outcomes of SARS-CoV-2 infections.

This study has several limitations that deserve discussion. First, some studies are still in the stage of clinical trials, and their indicator data reports are incomplete. Second, many types of studies were included, including cohort studies, randomized controlled studies, and case-control studies. Heterogeneity existed in data extraction due to different standards. Moreover, this study may increase the risk of various assessments due to different races, ages, severity of disease and underlying diseases (such as diabetes, HIV infection, and HBV infection).

More recent observational studies and clinical trials of coronavirus therapy, including 214 for lopinavir/ritonavir, are ongoing (available at http://covid19.trialstracker.net/). Detailed information is provided in Table 1. These trials will provide more evidence for the efficacy and safety of lopinavir/ritonavir or lopinavir/ritonavir combination regimens in patients with COVID-19. Through the discussion and analysis of this study, we provided some recommendations and suggestions for future clinical trials as follows: (1) high-quality RCTs of evidence-based medicine are urgently needed; (2) concurrent therapies, such as other antiviral agents or glucocorticoids, are strictly controlled or balanced; and (3) high-quality research designs, such as cluster-randomized control trials (CRCTs) or a stepped-wedge CRCT design, should be used as much as possible.

**Table 1:** Characteristics of the differences of age and gender in LPV/r therapy in patients with COVID-19.

**Study**	**Virus type**	**Country**	**Age**					**Gender**	
			**<65**		**>65**				
			**Cardiopulmonary disease**	**No cardiopulmonary disease**	**Cardiopulmonary disease**	**No cardiopulmonary disease**		**Male(%)**	**Female(%)**
Cao 2020	COVID-19	China	6	119	66	8		106(53.27%)	93(46.73)
Li 2020	COVID-19	China	3	17	11	2		17(51.52%)	16(48.48)
Young 2020	COVID-19	Singapore	2	7	8	1		9(50%)	9(50%)
Chen 2020	COVID-19	China	14	41	38	4		67(67.68%)	32(32.32%)
Jun 2020	COVID-19	China	8	22	20	2		36(69.23%)	16(30.77%)
Liu (1) 2020	COVID-19	China	2	1	6	1		4(40%)	6(60%)
Liu (2) 2020	COVID-19	China	12	18	21	5		31(55.36%)	25(44.64%)
Deng 2020	COVID-19	China	6	13	13	2		17(50%)	17(50%)
Wang 2020	COVID-19	China	15	78	38	4		72(53.33%)	63(46.67%)
Cai 2020	COVID-19	China	8	21	14	2		21(46.67%)	24(53.33%)

Cardiopulmonary disease: including Chronic Obstructive Pulmonary Diseases (COPD), Coronary heart disease (CHD) and hypertension.

Amber et al. found that the severity of COVID-19 largely depends on the patient's age [43]. Adults over 65 years of age represent 80% of hospitalizations and have a 23-fold greater risk of death than those under 65 years. We not only simply inhibited the virus but also effectively increased the immune responses of older people. In addition, Wang et al. also listed aging as a clinical challenge in patients with COVID-19 and concluded that older patients were more likely to develop poor outcomes and the severe form of the disease [44]. As research continues to develop, Gordan et al. identified biomarkers of biological age as predictors of COVID-19 disease severity [45]. For example, glycan diversity represents one of the main defenses of all higher organisms against pathogens and the repertoire of glycans changes with age. Thus, these researchers suggest that glycans should be the focus of biomarker discovery in COVID-19 cases. This information has greatly promoted the research progress of aging in COVID-19 and has great significance in clinical application. Pang et al. also agrees with the former, suggesting that altered receptor signals in aging and chronic disease play a role in COVID-19 infection and are associated with an increased risk of deterioration in different organs [46]. Thus, they concluded that aging may contribute to the deterioration of COVID-19 in older patients.

## CONCLUSIONS

This study comprehensively evaluated the efficacy and safety of LPV/r in the treatment of SARS, MERS and COVID-19. The purpose of this study was to analyze the data reported by previous studies on LPV/r in the treatment of SARS-CoV and MERS-CoV to explore the application value of LPV/r in the clinical treatment of COVID-19. This information offers guiding significance for the selection of first-line clinical treatments. Relevant studies have reported that LPV/r demonstrates good clinical efficacy in SARS, MERS and COVID-19 and does not increase the risk of adverse events compared to similar antiviral drugs. Therefore, its use should be promoted.

LPV/r exhibits good application value in the treatment of respiratory virus infections and HIV infection, and its antiviral effect is significant. Since the FDA approved the drug, LPV/r has played a substantial role in the treatment of respiratory virus and HIV virus infections. In the future, there will be more studies on the antiviral mechanism of LPV/r and new drug research and development in this field.

There will be more large-scale clinical trials of COVID-19 in the future. As these studies progress, researchers will gradually include aging as an indicator to be assessed. The difference in aging in COVID-19 treatment cannot be ignored now and in the future and will have a direct impact on mortality and virus clearance rates in older patients with COVID-19.

### Ethics approval and consent to participate

Our study does not contain data from any individual person or any animals.

## Supplementary Material

Supplementary Table 1

Supplementary Table 2
