# Supplementary material for: Comprehensive evaluation of the efficacy and safety of LPV/r drugs in the treatment of SARS and MERS to provide potential treatment options for COVID-19
Source: Aging (Albany NY). 2021 Apr 20;13(8):10833–52. doi: 10.18632/aging.202860 (PMC8109137; doi:10.18632/aging.202860)
Supplement: Supplementary Table 1 [file aging-13-202860-s001.docx]

Supplementary Table 1 Characteristics of included studies

Duration of

| Study/Author | Country | Research type | Virus type | Population | Age(year) | Gender(man/female) | | therapy(day) | Intervention | Comparison | J AD AD Score | NOS Score |
| --- | --- | --- | --- | --- | --- | --- | --- | --- | --- | --- | --- | --- |
| Chan 2003 | China | Matched cohort study | SARS | 75 | 43.23±14.34 | 25 | 50 | 10—14 | LPV/r 400/100 Q12H + standard treatment protocol  11/19 patientsreceived  ARVs:D4T/3TC/EFV = 3, | 977 matched controls from hospital data | 4 | 5 |
| Chen 2003 | China | Retrospective cohort  Case-control study with historical controls | SARS | 19 | 42.09±13.76 | 14 | 5 | >30 | d4T/3TC/NVP = 2,d4T/ddI/NVP = 3, Combivir/ EFV = 1, Indinavir/EFV =  LPV/r 400/100 Q12H as initial | N/A | 4 | 4 |
| Chu 2004 | China |  | SARS | 41 | 63.7±5.7 | 10 | 31 | 14 | therapy (n = 12),time of onset of symptoms 3.5 days. For rescue 138 patients received antivirals | 111 historical controls | 4 | 4 |
|  |  | Retrospective |  |  |  |  |  |  |  |  |  |  |
| Choi 2016 | Korea |  | MERS | 120 | 64.3±6.1 | 110 | 10 | 6 | among whom 120 received LPV/r- | N/A | 4 | 5 |
|  |  | observational study |  |  |  |  |  |  | containing |  |  |  |
| Alhumaid 2018 | Saudi Arabia | Retrospective observational study | MERS | 107 | 47.8±13.7 | 74 | 33 | N/A | 41 patients received LPV/r | N/A | 3 | 4 |
|  |  |  |  |  |  |  |  |  | *22* received PEP and *21* were not |  |  |  |
| Park 2019 | Korea | Retrospective case | MERS | 43 | 48.3±11.2 | 15 | 28 | *12* | given PEP; PEP protocol was RBV + LPV/r initiated between day 1 and | Historical controls from 4 hospitals | 4 | 4 |
|  |  | control study |  |  |  |  |  |  | day 3 after last unprotected exposure | located far apart |  |  |
|  |  |  |  |  |  |  |  |  | to the patient |  |  |  |
| Cao 2020 | China | Randomized tria | COVID-19 | 199 | 47.8±13.7 | 106 | 93 | *14* | 100 adult patients received LPV/r 400/100 Q12H | Supportive care alone | 4 | 6 |
|  |  |  |  |  |  |  |  |  |  | 16 received arbidol 7 received no |  |  |
| Li 2020 | China | Randomized trial | COVID-19 | 33 | 48±10.2 | 17 | 16 | *7—14* | LPV/r 200/500 Q12H | antivirals | 3 | 4 |
| Wang2020 | China | Case series | COVID-19 | 4 | 51±4.7 | 3 | 1 | *6—15* | LPV/r 400/100 Q12H | N/A | 3 | 3 |
|  |  |  |  |  |  |  |  |  | 5 patients treated with LPV/r |  |  |  |
| Young 2020 | Singapore | Retrospective cohort | COVID-19 | 18 | 47±16.0 | 9 | 9 | 1—3 | (200 mg/100 mgQ12H for up to 14 days) | N/A | 3 | 3 |
| Chen 2020 | China | Retrospective cohort | COVID-19 | 99 | 55.5±13.1 | 67 | 32 | 3—14 | LPV/r 500 mg Q12H | N/A | 4 | 6 |
| Jun 2020 | China | Retrospective cohort | COVID-19 | 52 | 49.7±10.0 | 36 | 16 | N/A | LPV/r Q12H for 5 days | ArdiboL: 34 patients. No  antivirals: 48 patients | 4 | 5 |
| Liu (1) 2020 | China | Retrospective cohort | COVID-19 | 10 | 43.2±16.5 | 4 | 6 | 5 | LPV/r 400/100 Q12H | N/A | 3 | 4 |
| Deng 2020 | China | Retrospective cohort | COVID-19 | 34 | 50.0±11.2 | 17 | 17 | 5—11 | LPV/r 400/100 Q12H | 16/33 patients also received arbido | 4 | 5 |
| Liu (2) 2020 | China | Retrospective cohort | COVID-19 | 56 | 53.1±9.7 | 31 | 25 | N/A | LPV/r 400/100 Q12H | N/A | 4 | 4 |
| Wang 2020 | China | Retrospective cohort | COVID-19 | 135 | 46.9±10.3 | 72 | 63 | N/A | LPV/r (dose not reported) | N/A | 4 | 6 |
| Cai 2020 | China | Comparative cohort study | COVID-19 | 45 | 48.9±7.9 | *21* | 24 | 14 | LPV/r 400/100 Q12H | Favipiravir | 4 | 5 |
| Guo 2020 | China | Comparative cohort study | COVID-19 | 8 | 56.1±11.2 | *7* | 1 | N/A | 947 patients received NNRTI- regimen 119 received LPV/r-based | HIV/AIDS patients in Wuchang and Qingshan distric | 3 | 4 |

SARS： Severe Acute Re-spiratory Syndrome ; MERS: Middle Eastern Respiratory Syndrome; COVID-19: Coronavirus Disease 2019; J AD AD Score <3(Low quality study),JADAD Score>3(High quality study)[Total Score:0-5]; NOS: Newcastle-Ottawa Scale ,NOS Score<4(Low quality study),NOS Score>4(High quality study) [Total Score:0-10],
