# Supplementary material for: Comprehensive evaluation of the efficacy and safety of LPV/r drugs in the treatment of SARS and MERS to provide potential treatment options for COVID-19
Source: Aging (Albany NY). 2021 Apr 20;13(8):10833–52. doi: 10.18632/aging.202860 (PMC8109137; doi:10.18632/aging.202860)
Supplement: Supplementary Table 2 [file aging-13-202860-s002.docx]

Supplementary Table 2. Clinical trials of LPV/ r against COVID-19 registered on ClinicalTrials.gov

| **Registration number** | **registration date** | **Location** | **Sponsor** | **phase** | **study type** | **intervention** |
| --- | --- | --- | --- | --- | --- | --- |
| ChiCTR2000029867 | 2020/2/15 | China | Beijing You'an Hospital, Capital Medical University | Phase 4 | Interventional | Carrimycin vs. lopinavir/ritonavir |
| NCT04261907 | 2020/6/2 | China | The First Affiliated Hospital of Zhejiang University | Phase 4 | Interventional | SC09 + ritonavir vs. lopinavir/ritonavir |
| ChiCTR2000029609 | 2020/2/6 | China | The Fifth Affiliated Hospital of Sun Yat-Sen University | Phase 4 | Interventional | Chloroquine vs. lopinavir/ritonavir |
| ChiCTR2000029603 | 2020/2/6 | China | The First Affiliated Hospital of Zhejiang University School of Medicine | N/A | Interventional | ASCO9 + Ritonavir vs. Lopinavir/ritonavir |
| ChiCTR2000029600 | 2020/2/6 | China | The Third People's Hospital of Shenzhen | N/A | Interventional | Favipiravir + interferon alpha vs. interferon alpha + Lopinavir/ritonavir + interferon alpha vs. interferon alpha |
| ChiCTR2000029548 | 2020/2/4 | China | The First Affiliated Hospital of Zhejiang University School of Medicine | N/A | Interventional | Baloxavir + Marboxil vs. Favipiravir vs.  Lopinavir/ritonavir |
| ChiCTR2000029541 | 2020/2/3 | China | Zhongnan Hospital of Wuhan University | N/A | Interventional | Darunavir + Cobicistat vs. Lopinavir/ritonavir |
| ChiCTR2000029539 | 2020/2/3 | China | Tongji Hospital, Tongji Medical College, Huazhong University of Science and Technology | N/A | Interventional | Lopinavir/ritonavir |
| ChiCTR2000029468 | 2020/2/2 | China | Institute of Emergency Medicine and Disaster Medicine Sichuan  People's Hospital, Sichuan Academy of Medical Sciences | Phase 4 | Interventional | Lopinavir/ritonavir + emtricitabine + tenofovir vs. lopinavir/ritonavir |
| ChiCTR2000030218 | 2020/2/25 | China | The Fifth People's Hospital of Ganzhou | N/A | Interventional | Lopinavir/ritonavir (+ Tradtional Medicine) |
| ChiCTR2000029308 | 2020/1/23 | China | Wuhan Jinyintan Hospital (Wuhan Infectious Diseases Hospital) | N/A | Interventional | lopinavir/ritonavir |
| ChiCTR2000030703 | 2020/3/10 | China | Xiangya Hospital of Central South University | N/A | Interventional | Ixekizumab + up to three of interferon-alfa, lopinavir/ritonavir, chloroquine, ribavirin, umifenovir |
| ChiCTR2000030187 | 2020/2/24 | China | Jingzhou First People's Hospital | N/A | Interventional | Lopinavir/ritonavir |
| NCT04328012 | 2020/3/27 | United States | Bassett Healthcare | Phase 2/Phase 3 | Interventional | Lopinavir/ritonavir vs. hydroxychloroquine vs. ARB (Losartan) |
| EUCTR2020-001156-18-ES | 2020/3/4 | Spain | Fundacion para la Investigacion Biomedica Hospital Universitario LaPaz (FIBHULP) | Phase 3 | Interventional | Dolquine, Azithromycin, Lopinavir/ritonavir |
